# Immorally obtained principal increases investors’ risk preference

**DOI:** 10.1371/journal.pone.0175181

**Published:** 2017-04-03

**Authors:** Chuqian Chen, Jiaxin Chen, Guibing He

**Affiliations:** 1 Department of Social Work and Social Administration, the University of Hong Kong, Pokfulam, Hong Kong SAR, P.R. China; 2 Department of Psychology and Behavioral Sciences, Zhejiang University, Hangzhou, P.R. China; Middlesex University, UNITED KINGDOM

## Abstract

Capital derived from immoral sources is increasingly circulated in today’s financial markets. The moral associations of capital are important, although their impact on investment remains unknown. This research aims to explore the influence of principal source morality on investors’ risk preferences. Three studies were conducted in this regard. Study 1 finds that investors are more risk-seeking when their principal is earned immorally (through lying), whereas their risk preferences do not change when they invest money earned from neutral sources after engaging in immoral behavior. Study 2 reveals that guilt fully mediates the relationship between principal source morality and investors’ risk preferences. Studies 3a and 3b introduce a new immoral principal source and a new manipulation method to improve external validity. Guilt is shown to the decrease the subjective value of morally flawed principal, leading to higher risk preference. The findings show the influence of morality-related features of principal on people’s investment behavior and further support mental account theory. The results also predict the potential threats of “grey principal” to market stability.

## Introduction

Conflicts between morality and interest are ubiquitous for governments, corporations, and individuals. Sometimes, such conflicts end with venality, creating “dirty money”. Although dirty money is hated and despised, large amounts of it are currently circulating in the world’s capital markets. As estimated by the IMF in 2015, money laundering accounts for more than 5% of global GDP, and money laundering mainly occurs through investments. Thus, the stability of global and local markets would be threatened if individuals were less prudent and more risk-seeking when investing dirty money. However, it remains unclear how the morality of principal obtaining means impacts investors’ risk preferences. To fill in this knowledge gap, this research attempts to combine perspectives from both risky decision making and moral psychology.

### Preferences in risky decision making

Many studies of risky decision making have focused on individuals’ investment choices. The identified impact factors mainly fall into three categories: the characteristics of decision makers, features of options, and contextual clues. Decision makers’ relevant characteristics that matter include their age[1], gender[2], regulatory focuses[3], and cultural backgrounds[4]. However, emotions[5], construal level[6], self-control resources[7], and feelings of power[8] at the moment of choice are also important factors. Outcome valence, frameworks[9], probabilities of gains and losses in risky options[10], the amount of money involved[11] and default choices are all features of investment options that have been considered. Contextual clues, such as time pressure[12], outcomes of previous decisions[13], and hints of past wins[14], can also influence individuals’ investment choices.

However, few studies have focused on moral elements or, in particular, on the morality of the means with which the principal was acquired. In general, venture investments are trade-offs between less risk of losing capital and the chance to earn larger profits[9]. It is therefore highly possible that individuals’ investment choices will change if their attitudes toward capital are altered by the morality of the means by which their capital was obtained.

### The fungibility of money

There are conflicting opinions regarding whether individuals use money differently based on its source. The economic assumption of fungibility argues that the source of money makes no difference in its consumption[15]. Conversely, mental account theory[16] contends that people group income into various mental accounts (such as regular income or windfall income), and that money in these distinguished mental accounts is not fungible. Accumulating evidence is in line with the latter assumption. People’s money consumption varies depending on whether money is paid to them before or after they provide service to others, the money-exchanging route (refunded from tokens or coins)[17], and the receiver’s emotion when the money is acquired[18]. In gambling, people take more risks when they play with previous gambling gains than with their normal income[19]. During online shopping, consumers spend windfalls more generously than money from other sources[20]. Therefore, it is also possible that the morality of the source of capital influences individuals’ attitudes toward capital, which in turn changes how they invest that capital.

### The impact of immoral obtaining means

Money can acquire moral meaning based on its source[21]. People tend to distance themselves from money with immoral associations and underestimate its value, just as they treat other items with moral flaws.

Typically, individuals distance themselves from items relating to immoral persons or immoral behaviors to avoid “moral contamination” [22]. Students are unwilling to wear evil persons’ shirts [23]. Participants wash their hands for a longer time after shaking hands with a liar, or even after holding photos of a misbehaving person’s hands[24]. Self-cleansing also occurs when people themselves behave immorally[25, 26]. Such purposeful distancing is also apparent with money. According to Carruthers and Espeland [21], the meaning of money can come from its proximate source, and money earned through disgraceful behaviors has immoral meanings. When participants blame money for a man’s immoral behavior, they wash their hands longer after touching cash[24]. After imagining earning money by immoral means, participants attempt to connect less with the money and thus plan to spend less of that money on travelling to lessen such connection[27].

When objects are considered immoral, they are less desirable, and their value can be corrupted, which can be reflected in customers’ hesitation[28] and lower bid prices[29] when buying products from companies known to be morally questionable. Similarly, people are prone to undervaluation when confronting immoral money. For example, students consider a $50 participant fee from an unscrupulous enterprise to be less valuable than a $50 participant fee from a morally neutral company[22].

Based on past findings, we believe that individuals who earn principal by unethical means will undervalue their principal and attempt to distance themselves from it. Therefore, they will care less regarding the risk of losing capital, or may even desire to lose it to create distance. Both mechanisms ultimately involve risker investment choices.

### Guilt: A moral emotion

As a moral emotion, the feeling of guilt plays an essential role in forming people’s attitudes and behavioral tendencies toward immoral objects.

Moral emotions are emotions “that are linked to the interests or welfare either of society as a whole or at least of persons other than the judge or agent” [30]. These emotions mediate the relationship between moral values and moral judgements and will influence moral behaviors[31–33], as a result.

As one of the most important moral emotions, guilt arises when a moral transgression is one’s own responsibility, the responsibility of individuals to whom one is closely related [34], or the responsibility of an organization to which one belongs [35]. Guilt drives individuals and organizations to atone for their wrongdoings[36–38]. More specifically, guilt has been shown to be the cause for cleansing after immoral behaviors[25, 26, 39]. The authors believe that guilt may also result in individuals’ intentions to distance themselves from immorally earned money and devalue it. Therefore, guilt would play a mediation role between the morality of the means by which capital is obtained and the riskiness of individual investments involving such capital.

### The present research

**Fig 1:**
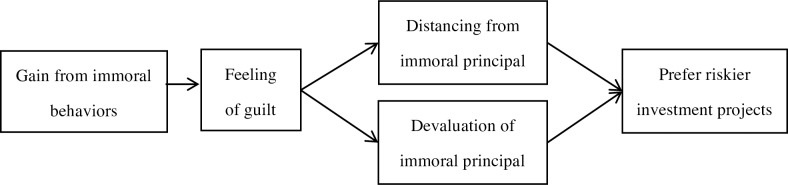
Hypothesized mechanism in this research.

Based on past studies, we assume that people feel guilty after they earn capital immorally. Guilt would lead people to get away from contaminated principal and make people regard such principal as less valuable. Thus, people tend to risk this capital more in investments because increased risk may help them distance themselves from the capital through loss, and the pain of loss is lower when the subjective value of capital drops (Fig 1). If this assumption is true, investors will be more risk-seeking only when their capital is obtained through guilt-causing behavior but not when they invest with neutral money after feeling guilty about something else.

Three studies were conducted to test the hypothesis. In study 1, the effect of principal-source morality on investors’ risk preference and its boundary condition were tested. Study 2 examined the mediating effect of guilt. In studies 3a and 3b, two possible paths linking feelings of guilt to higher risk preferences were examined, and new immoral money-earning situations were included to increase external validity. The morality of principal earning means was manipulated via real tasks in the first two studies and by imagination in study 3.

## Ethics statement

The current research was approved by the Research Ethics Board of Department of Psychology and Behavioral Sciences at Zhejiang University. All participants in study1 and study 2 read a consent form on paper and gave verbal confirmation before the experiment. The participants in study 3 read their consent form on a computer screen and clicked “confirm” before the experiment.

## Study 1 The impact of principal source morality on investors’ risk preferences

In study 1, we aimed to uncover the impact of the morality associated with the means by which principal was earned (hereafter, principal source morality) on investors’ risk preference. Whether that influence generalizes to investments with moral principal after immoral behaviors was also examined. A 2(morality of description: moral/immoral)*2(principal’s relevance to description: relevant/irrelevant) between-subjects design was used. The participant’s choice between the two investment projects was the dependent variable.

### Methods and materials

The sample of this study consisted of 126 (46 males, age *M* = 20.87, *SD* = 1.84) undergraduate and graduate students at Zhejiang University who were recruited from the campus BBS. Each participant received at least ¥10 as a participant fee.

When the experiment began, each participant repeated a boring action for 10 minutes. (In pilot study 1, 67(age *M* = 20.36, *SD* = 2.35, 35 males) undergraduate students rated how boring 17 tasks were. This task scored 3.87 out of 5(very boring) and was rated the most boring of all tasks). The participants moved rice grains one by one from bowl A to bowl B continuously without reading the time or taking rests during the task. Each participant earned ¥10 for the operation task. Next, the participants rated how interesting the task was on a 9-point scale (-4 = extremely boring, 4 = extremely interesting, 0 = neutral). Then, the participants described the task’s interestingness in three sentences following the experimenter’s instructions.

Instructions for the moral description group consisted of the following:

Your descriptions will be used to help recruit future participants. We will show descriptions of feelings from previous participants to potential participants to help them decide whether to join the experiment. Please write 3 sentences in your own words to describe your feelings about the interestingness and attractiveness of the operation task. Please write down these descriptions in the “Description of Feelings” part on the next page. Make sure that your descriptions are matter-of-fact (there are no right or wrong answers, please write down your true feelings).

Participants in the immoral description group read the following instructions:

Your descriptions will be used to help recruit future participants. We will show descriptions of feelings from previous participants to potential participants to help them decide whether to join the experiment. Therefore, we need you to describe the operation task as interesting and attractive. We hope you can write based on our request, no matter what your true feelings are. There are 10 sentences listed below, and they all describe the operation task as very interesting and attractive. Make sure you choose 3 from the descriptions provided and copy them to the “Description of Feelings” part on the next page.

**Fig 2:**
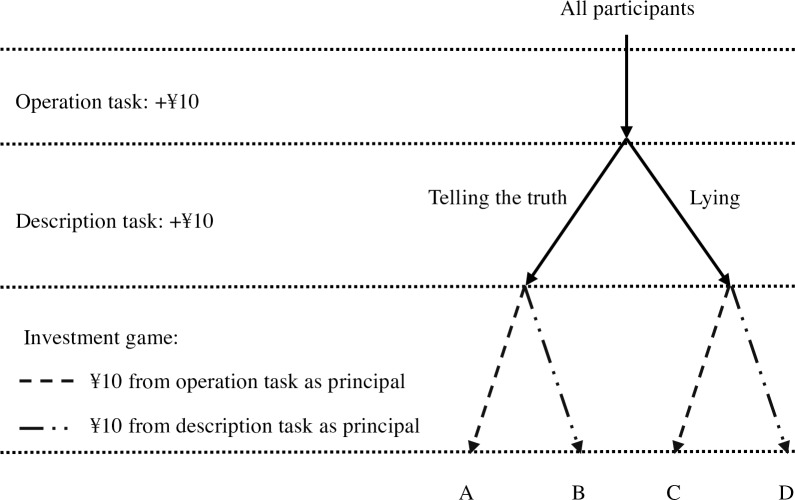
Procedure of study 1.

Descriptions provided for the immoral group include “it feels like an interesting game, and I couldn’t stop playing it!”, and “it was so interesting that I almost forgot the time!”. The participants were required to sign under their descriptions to become more involved. In fact, no one except the experimenter would read the descriptions. Each participant earned ¥10 for finishing the description (and thus earned ¥20 in total), whether they lied per the experimenter’s request or wrote their truth feelings in the description. During the experiment process, participants were informed via text each time they earned a sum of money, and their total income was summed and cashed together when the experiment finished). They then put ¥10 out of their ¥20 into an investment game (Fig 2). Participants in the description-irrelevant principal group used the ¥10 from the operation task (irrelevant to moral or immoral behavior) as principal, and those in the description-relevant principal group used the ¥10 from the description task (direct gain from moral or immoral behavior). The game was designed to imitate real investments in the market (In pilot study 2, titration was used to find the equivalent point in a steady investment project. The study’s participants included 65(age *M* = 20.42, *SD* = 2.36, 22 males) undergraduate students who rated the risk level of the risky investment project as between “low” and “medium”). The investment game was as follows:

Principal: ¥10
Investment options:
A Steady project: **100**% to gain ¥13 (principal + profit = ¥23)
B Risky project:
  **72**% to gain ¥25 (principal + profit = ¥35)
  **10**% to gain ¥0 (principal + profit = ¥10)
  **18**% to lose ¥10 (principal + profit = ¥0)

Every participant cashed his or her remaining ¥10 immediately after the choice. They were then debriefed about the deception. No one reported any suspicion. Ten participants were randomly chosen two weeks after the experiment to receive their gains from the investment game. The chosen participants who picked the steady project in the previous investment game received ¥23. Furthermore, 72%, 10%, and 18% of those who picked the risky project received ¥35, ¥10, and ¥0 respectively. The subjects were informed in advance about the arrangement but were not clear about how many of them would be chosen to receive the gains.

### Results

All participants in the immoral description groups copied the sentences in the description task per the experimenter’s instructions. The operational definition of lying in this research is describing the task as interesting although the participant thought it was boring. Therefore, 12 participants in the immoral description groups (C and D in Fig 2) who initially rated the operation task as interesting (rated interestingness greater than 0) and were then asked by the experimenter to describe the task as interesting were excluded. To ensure the comparability among groups, 17 participants in the moral description groups (A and B in Fig 2) who considered the operation task interesting were also excluded. A total of 97 participants remained (all of whom rated the operation task as not interesting, 43 lied in the description task and 54 told the truth, age *M* = 20.90, *SD* = 1.69, 37 males). The descriptions from those in the moral description groups were consistent with their ratings. There were no significant differences of age or gender among the 4 groups. The participants from the various groups differed in their ratings of interestingness (there was an interaction of morality of description and principal’s relevance to description, *F* = 4.201, *df* = 1, *p* = .043). The results did not change when the subjective interestingness of the operation task was included as a covariate.

**Fig 3:**
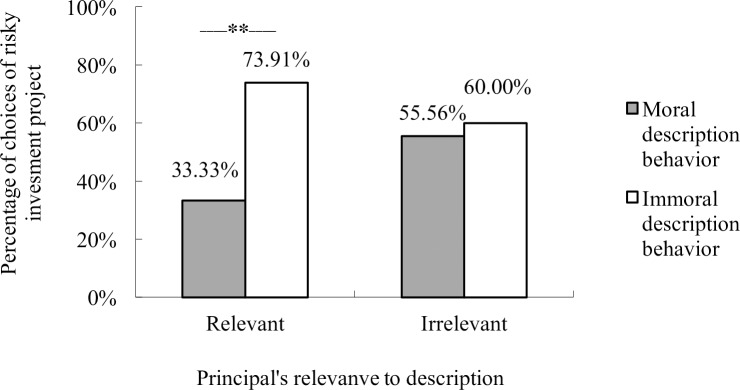
Interaction of morality of description and principal’s relevance to description on investment choice.

Significantly more people who lied chose the risky investment project than those who told the truth (*χ*^*2*^ = 5.108, *df* = 1, *p* = .026). The main effect of the principals’ relevance to description was not significant (*χ*^*2*^ = .290, *df* = 1, *p* = .684). With gender, age, and the subjective interestingness of the operation task as covariates, logistic regression revealed a significant interaction of the two independent variables (*p* = .048, Table 1). A simple effect analysis showed that participants who lied preferred the risky project more than those who did not only when their principal was gained from description (between B and D in Fig 2, *χ*^*2*^ = 8.194, *df* = 1, *p* = .005, *Φ* = .320; logistic regression with covariates *B* = -1.793, *p* = .009). There was no difference between the two groups that invested with gains from the operation task (A and C in Fig 2, *χ*^*2*^ = .093, *df* = 1, *p* = 1.000, *Φ* = .044; logistic regression with covariates *B* = .377, *p* = .588, Fig 3). In addition, there was no significant difference between any other two groups.

**Table 1:** Results of the logistic regression.

	**B**	**S.E.**	***df***	***p***
Morality of description	-3.614**	1.489	1	.015
Principal’s relevance to description	-2.589*	1.539	1	.093
Morality of description*Principal’s relevance to description	1.848**	0.936	1	.048
Subjective interestingness of operation task	0.006	0.215	1	.979
Gender	-1.056**	0.516	1	.041
Age	0.23	0.148	1	.119
Constant	2.376	3.732	1	.524

*.05≤p < .1

**.001≤p < .05

*** p < .001; morality of behavior: 1 = immoral, 2 = moral; source of principal: 1 = relevant, 2 = irrelevant; investment choice: 1 = steady investment project, 2 = risky investment project; gender: 1 = male, 2 = female

### Discussion

Study 1 shows that people are more risk-seeking in investments when their principal is obtained immorally. Indeed, investors’ risk preferences do not change when they invest with money earned by neutral means even after engaging in deceptive actions. These results are in line with our hypothesis. However, the difficulty of obtaining principal may work as a confounding variable because those who lied earned principal by copying descriptions, whereas those who told the truth were required to organize descriptions on their own. Thus, it is possible that difficulty in obtaining principal influences investors’ conservatism. We attempted to rule out this explanation in study 2.

## Study 2 The mediating effect of guilt

Study 2 aimed to test the mediating effect of guilt and to simultaneously control the difficulty of obtaining principal as a confounding variable. Participants in the two groups invested with direct gains from moral or immoral behaviors, and reported their guilt before investment. Unlike study 1, the two groups with morality-irrelevant capital were not included in this study. Context-related emotional experiences other than guilt were measured and analyzed as covariates to remove their influence on outcomes. Participants’ reflective moral attentiveness (the extent to which the individual regularly considers moral matters) was also measured as a covariate such that individuals’ processing depth of moral-related information could be controlled in both groups.

### Methods and materials

Study 2 included 91 students from the Zhejiang University BBS who voluntarily joined. The payment rules were the same as those in study 1.

**Fig 4:**
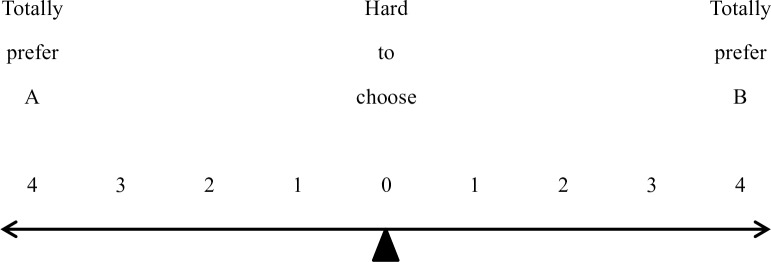
Measurement of investment preference.

Each participant first took part in a 10-minute boring task on a computer. They repeatedly moved a sliding block from one end of a line to another on the screen with a mouse and earned ¥10 for performing that task. The participants then rated the interestingness of the operation task and were asked to lie or tell the truth in a description following the task. They were told that their descriptions would be read by potential participants in the future and that they would earn ¥10 for their description. Unlike study 1, the participants in both groups chose and copied 3 out of 15 sentences provided by the experimenter to even out the difficulty of the description task. For the immoral principal group, all 15 sentences described the operation task as extremely interesting and attractive. For the moral principal group, the 15 sentences provided descriptions ranging from extremely boring to extremely interesting, and the participants could choose what best expressed their feelings. Following this task, participants rated how strong their feelings were (“angry, happy, tired, shy, guilty, calm, confident, sad, surprised and anxious”) on a 7-point scale (1 = not strong at all, 7 = extremely strong). Then, they joined the same investment game (with ¥10 from the description task as principal) as in study 1. However, instead of making choices, the participants each circled a number in a picture (Fig 4) to express their relative preference for investment projects.

The participants finished the reflective moral attentiveness scale (see S1 File) by Reynolds [40] after the game and were then paid ¥10 each. Eight participants were randomly chosen one week after the experiment to receive their gains from the investment game. Subjects were informed in advance about the arrangement but were not clear about the proportions.

### Results

Twelve participants were excluded for rating the operation task as interesting or showing doubt regarding the situation. Thus, 79 (30 males, age *M* = 20.68, *SD* = 2.50) participants remained. The two groups were not significantly different in age, gender composition, or in the subjective interestingness of the operation task.

**Fig 5:**
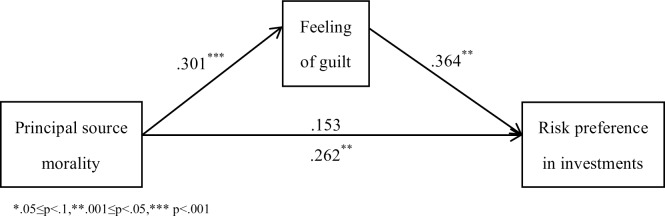
Full mediation effect of guilt.

The numbers that the participants circled in investment game were converted into scores from -4 (completely prefer steady investment project) to 4 (completely prefer risky investment project). They were then normalized via the Bloom method based on their rank in SPSS because they did not comply with a normal distribution. A t-test revealed that individuals who invest with immoral principal (*M* = .27, SD = .94) prefer the risky project more (*T* = 2.72, *df* = 77, *p* = .008, Cohen's *d* = .62) than those investing with moral principal (*M* = -.29, *SD* = .86). Ratings of the emotional experiences and scores on the reflective moral attentiveness scale also did not fit the normal distribution. A rank sum test showed that individuals who lied experienced more anger (*p* = .050), shyness (*p* = .002), surprise (*p* = .012), anxiety (*p* = .008), and guilt (*p* = .000), and less calm (*p* = .032) than those who told the truth. Participants in the two groups did not differ in their reflective moral attentiveness (*p* = .741). Bootstrapping (*N* = 5000, see S2 File) method [41] was used in the mediation test. Normalized ratings of anger, shyness, surprise, anxiety, calm and reflective moral attentiveness (Cronbach's alpha = .798) were entered as covariates altogether. 95% CI of the indirect effect was (.0338, 1.1126), and 95% CI of the direct effect was (-.4620, 1.7853). Therefore, feelings of guilt fully mediate the relationship between principal source morality and investors’ risk preferences (Fig 5). No mediation effect was found for any other emotions.

## Discussion

Study 2 replicated the impact of the morality of the source of principal on investors’ risk preferences after controlling for the difficulty of obtaining the principal and the depth of assessing moral-related information. More importantly, a full mediation effect of guilt was revealed: individuals with immoral principal were guiltier, and they risked more in their investments as a result.

Participants in the immoral group were forced to behave unethically to earn their principal. As the results show, negative emotions were aroused, including anger, surprise, and anxiety. Analyzing these emotions as covariates makes the explanation via guilt more convincing.

Both study 1 and study 2 took lying as the example of an immoral principal source, which may undermine the external validity. Thus, a new situation was introduced in study 3.

## Study 3 The mechanism of guilt’s impact on risk preference

Study 3 aimed to examine two mechanisms of how guilt leads to higher risk preference: the desire to reduce guilt by distancing oneself from immoral capital, and the decreased subjective valuation of immoral capital. These mechanisms would be tested in two different contexts (3a and ab). Due to concerns regarding research ethics, manipulation was achieved using imagination in this study.

### Study 3a

#### Methods and materials

Study 3a employed a sample of 80 students at Zhejiang University. Each participant received a ¥5 participant fee. The entire procedure was conducted in the Visual Basic program.

The participants first completed an imagination task. Sentences creating a complete story appeared one by one on the screen. Participants read each sentence carefully and imagined themselves as the leading character in that story.

The story of the [moral/immoral] principal group proceeded as follows:

You were invited to join a psychological experiment involving a one-hour operation task. The experiment was extremely boring and you eventually finished it. When you finished, the experimenter gave you a present as a reward. While sending you away, the experimenter received a phone call. The experimenter talked on the phone for a while and came to you, covering the mobile receiver. The experimenter told you that the caller was a student interested in the experiment. That student was not sure whether to join the experiment, so the student wanted to know the feelings of past participants. The experimenter asked you to describe [your true feelings to help the calling student decide/ the experiment as extremely interesting to attract the calling student to join] and promised to give you ¥10 for doing so. You described [your truthful feelings on the phone and advised the calling student not to come/ the experiment as extremely interesting on the phone as requested by the experimenter]. After you hung up, the experimenter gave you ¥10.

Each sentence lasted for 10 seconds on the screen, so the entire imagination task lasted 100 seconds. After the story, the participants were asked how much they received as the leading character in the story. Then, the participants rated their guilt about earning the ¥10 on a 9-point scale (1 = not guilty at all, 9 = extremely guilty). The students continued to imagine using the earned ¥10 to invest (the same game as in studies 1 and 2) and chose the project they preferred. Then, they ranked 4 goals (“to ensure gains”, “to maximize gains”, “to reduce guilt” and “others”) according to each goal’s importance in deciding which investment project to choose (1 = most important, 4 = least important). All participants then spent 30 seconds reading the entire story again to reinforce their memory. Finally, pictures showing a pin, a card case, a paper cup, and a lollipop appeared on the screen in turn, and the participants estimated how many objects in the picture the ¥10 principal could buy, one after another. Before leaving, the participants were paid and then were asked to guess the experiment’s aim.

#### Results

No one correctly guessed the aim of the experiment. Twelve participants were removed because they failed to answer the question after the imagination game. Thus, 68 participants (18 males, age *M* = 23.32, *SD* = 4.17) remained. Individuals with immoral earnings were guiltier (Mann-Whitney U test, *p* = .000) and were more likely to prefer the risky investment project (64.7% vs. 35.3% to choose project B, *χ*^*2*^ = 5.882, *df* = 1, *p* = .028, *Φ* = .294) than those with moral earnings.

We subtracted the rank of each goal from 5 to obtain the goal’s relative importance for making investment decision. Larger numbers indicate higher importance (Table 2). The relative importance of none of the 4 goals differed between the 2 groups (Mann-Whitney U test). A two-step bootstrap (N = 5000, mediator 1 = feeling of guilt, mediator 2 = relative importance of “to reduce guilt”, see S2 File) analysis [41] revealed that motivation to reduce guilt was not the reason for choosing the risky investment project after feeling guilty (95% CI of the indirect effect was (-.0439, .3194)).

**Table 2:** The relative importance of every goal.

**Goal**	**3a**		**3b**	
	Moral principal group	Immoral principal group	Moral principal group	Immoral principal group
To ensure gains	3.26 (.99)	2.97 (.97)	3.33 (.96)	2.86 (1.03)
To maximize gains	2.71 (1.00)	2.97 (1.06)	2.89 (1.06)	13.03 (.92)
To reduce guilt	1.97 (.80)	2.29 (2.29)	1.94 (.92)	1.80 (1.02)
Others	1.97 (1.14)	1.76 (1.05)	1.83 (.78)	2.29 (1.10)

The estimated numbers of objects the principal could buy (Table 3) did not follow a normal distribution. The study constructed a subjective value index by first normalizing and standardizing the numbers all participants given to each object (to remove differences caused by the objects’ characteristics), and then added up each participant’s 4 transformed numbers. The index of immoral principal (*M* = -.23, *SD* = 2.09) does not differ from that of moral principal (*M* = .21, *SD* = 2.74). A two-step mediation effect of guilt and the principal’s subjective value was significant (bootstrap, *N* = 5000, mediator 1 = feeling of guilt, mediator 2 = subjective value index, 95% CI was (.0622, .9902), see S2 File). Thus, individuals who lied to earn the principal are guiltier, so they consider the principal as less valuable and therefore more readily invested it in riskier projects.

**Table 3:** Estimated numbers of objects principal can buy.

**Objects**	**3a**		**3b**	
	Moral principal group	Immoral principal group	Moral principal group	Immoral principal group
Pin	156.65 (269.80)	120.38 (178.94)	161.06 (231.98)	76.29 (48.39)
Card case	51.15 (173.92)	8.09 (11.38)	14.92 (26.49)	9.83 (11.72)
Paper cup	25.56 (31.16)	24.59 (37.31)	31.28 (41.54)	26.17 (25.65)
Lollipop	13.71 (16.42)	12 (8.84)	14.39 (9.78)	16.89 (19.15)

### Study 3b

The sample in study 3b consisted of 80 Zhejiang University students. All steps (imagination task, imagination check, rating of guilt, imagined investment, sorting of goals based on their importance, estimation of principal’s value) and materials were the same as 3a, except for the imagined stories.

The story for the [moral/ immoral] principal group was as follows:

You were in line at the checkout counter in a convenience store near your school, it was crowded in the store and the line was very long. M, a customer who had just checked out, hurried out of the door. You saw that M had not zipped up his pocket, and a pile of money fell out of his pocket. M did not notice it, and there was no other witness when you looked around. You left the line and exited the store to pick up the money [and returned it to M. M was very grateful, and M pulled ¥10 out of the pile to give you as a reward. M insisted that you take the money before M left, and you finally took it /prepared to return it to M. Before catching up with M, you pulled ¥10 from the pile and kept it yourself. When you stopped M and gave M the remaining money, M was not suspicious and appeared grateful].

Nine participants were excluded for failing the question after imagination. Thus, 71 (32 males, age *M* = 21.44, *SD* = 2.30) participants remained. The analytical steps in study 3b mirrored those in study 3a. Participants with immorally gained principal were more likely to choose the risky investment project (65.7% v.s. 38.9%, *χ*^*2*^ = 5.117, *df* = 1, *p* = .033, *Φ* = .268), and they also felt guiltier (Mann-Whitney U test, *p* = .000).

The relative importance of each of the 4 goals was compared between the 2 groups, and no differences were found. A two-step mediation effect with feelings of guilt and the relative importance of reducing guilt as mediators was not significant (bootstrap, N = 5000, 95% CI = (-.1309, .6335), see S2 File).

The subjective value index for immorally earned principal (*M* = -.34, *SD* = 2.49) did not different from that of morally earned principal (*M* = .30, *SD* = 2.73). A two-step mediation effect of feelings of guilt and the principal’s subjective value index as mediators was significant (bootstrap, N = 5000, 95% CI = (.0135, .9705), S2 File).

### Discussion

**Fig 6:**
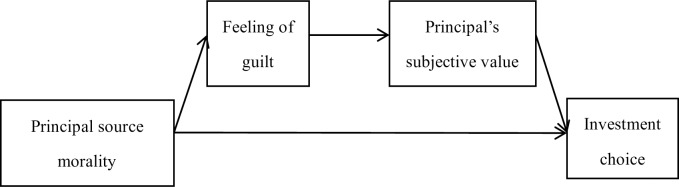
Schematic diagram of two-step mediation effect in study 3.

With 2 parallel studies, study 3 replicated the effect of the principal source morality in a new context with a new manipulation method. More importantly, it revealed the mechanism behind the impact of guilt on investment choices: guilty individuals undervalue their immorally earned principal, such that they will risk it more readily for a larger profit (Fig 6). In contrast with one of the former hypotheses, the assumption of reducing guilt by distancing oneself from immorally earned principal was not proven.

The effect size of study 3a and 3b (both small) was smaller than those in the previous 2 studies (both medium). The authors believe this result was caused by the weaker effect of manipulation by imagination than manipulation by a real task.

## General discussion

### Main results

Study 1 revealed that investors risk more when their principal is earned unethically, and their risk preferences do not change when they invest with morally earned capital after engaging in immoral behavior. Study 2 showed that feelings of guilt fully mediates the relationship between the principal source morality and investors’ risk preference. Employing 2 sub-studies, study 3 found that guilt increases investors’ risk preference by reducing the subjective value of immorally earned principal. The study also generalized its findings to new contexts based on a new manipulation method.

Nevertheless, the hypothesis regarding people’s motivation to distance themselves from immoral principal by choosing the risky project was not proven. In our opinion, it is possible that people perceive gains from investments as distinct from principal. Therefore, principal will be distanced as long as they invest regardless of the project they choose, which is similar to money laundering. It is also possible that all people care about during investments is the best monetary outcomes, and they engage in other efforts to relieve guilt before or after investing.

### Impact of principal source morality on risk preference

The findings in this article support mental account theory, which emphasizes that the consumption of money can be affected by differences in the way in which it is acquired. In this research, the difference lies in moral meaning. According to the classical definition of mental account[16], principals with various moral meanings do not belong to distinct accounts, particularly when they are both given by the experimenter as a reward for descriptions and when the difficulty of descriptions is matched. This research adds moral meaning as a new dimension of classification in mental accounts. It also reveals that mental accounts distinguished by the morality of the source of capital affect investments via feelings of guilt and subjective values.

In this research, the impact of immorally earned principal does not generalize to investments with moral principal after unethical practices. This contrasts with research by Xie et al[24], in which participants held negative attitudes toward all money because they blamed certain money for causing misbehaviors. In our opinion, this result is grounded in the difference between “money as cause” and “money as outcome”. When money causes evil behaviors, any money of the same (or larger) amount may have the same effect such that all money is considered evil. By contrast, when money acquires immoral meanings because it is a direct gain from certain moral transgressions, money unrelated to that particular misconduct remains clean. Therefore, investment behaviors with clean money will not change.

### Impact of guilt on risky decision making

Combining past research with the present studies, we propose that guilt’s influence on risky decision making can be affected by whether the cause of guilt and the ensuing decision making derive from the same context.

When the cause of guilt is unrelated to subsequent choices, guilt works on a general level. Based on appraisal tendency theory [5], Kouchaki, Oveis, and Gino[42] found that guilty people take more risks in investments because feeling responsible for something bad increases their sense of control. This type of context-independent guilt did not play a role in our research because those who invested with irrelevant money after lying in study 1 did not change their risk preference. In our opinion, this result is because participants behaved immorally at the experimenter’s behest rather than purely of their own volition in this research, such that their sense of control was not enhanced.

When the cause of guilt and decision making derive from the same context, the effect of guilt are closely linked to specific features of the context. Mancini and Gangemi[43] conducted research in which participants felt guilty for an imagined traffic violation before choosing from two fining options with different risks. Guilt increased their desire to be punished, so they tended to process information in a loss framework, which leads to riskier choices[9]. Similarly, guilt in this research plays its role in a context-relevant way: by influencing the subjective value of gains from guilt-inducing behaviors. Under these circumstances, researchers must analyze in a “context-sensitive” manner to understand the impact of guilt.

### Contributions and implications for future researches

Theoretically, this research adds to the moral dimension of capital as an influential factor on individual’s investment behavior. Both the impact of principal source morality and its mechanism are revealed in this study. In addition, this research challenges the assumption of money’s simple fungibility and further supports mental account theory. The findings from this research also suggest a new way to analyze the role of emotions in decision makings. As a practical matter, the outcomes indicate that a capital market with a high percentage of immorally gained principal may experience more risky investments. These investment preferences might produce economic vitality in the short-term while posing a threat on the long-term healthy, stable and sustainable economic development. Therefore, it is important to economic growth to reduce the percentage of immoral principal and increase the percentage of moral principal via more effective financial supervision of the capital market.

Despite the significances of this study, it nonetheless has its limitations. First, all participants were students who had little experience with investments, and they invested in a game with a maximum gain of ¥35. Studying experts in real investments would results in more convincing outcomes. Second, participants in the immoral principal groups were forced to behave badly or to imagine doing so. Conceptually, this is different from actively choosing to profit from immoral behaviors. Therefore, the present findings cannot be widely generalized. In study 3b, the participants imaged taking the initiative to behave immorally, and their investment and its mechanism did not differ from their peers in study 3a. Although this may imply that those two circumstances share some similarity with regard to risky investments, more evidence from strictly manipulated experiments are needed before any conclusions can be drawn. Whether investors’ risk preferences will change when they freely choose to earn their principal immorally is worth exploring. Third, investors’ risk preference would be affected not only by morality of principal obtaining means and guilt, but also by traits such as risk tendency and sense of morality. Although the latter was ruled out to some extent by random groups and covariates in the analysis, it is worth exploring in the future how risk preference and sense of morality as characteristics can moderate the effect of principal source morality. Finally, the moral meaning of money can be influenced in more ways [21] than how it is earned. Paying more attention to other dimensions such as whom it is received from, by what kind of government it is issued, and where would the gains to investment flow and comparing the effects of these dimensions with the means of obtaining money seems a promising direction for future research.

## Conclusions

Employing three studies, this research proves that people engage in riskier investments when their principal is earned immorally. Feelings of guilt caused by immoral means of earning corrupts the value of that principal and increases people’s willingness to risk it for larger gains. This effect is stable across various contexts and different methods of manipulation. However, this effect disappears when people invest with neutral principal after engaging in wrongdoing.

## Supporting information

S1 File Reflective moral attentiveness scale. (PDF)

S2 File Bootstrap results in study 2 and 3. (PDF)

S3 File Minimum data set. (PDF)
