# Correction: Immorally obtained principal increases investors’ risk preference

**DOI:** 10.1371/journal.pone.0178892

**Published:** 2017-05-26

**Authors:** Chuqian Chen, Jiaxin Chen, Guibing He

There are errors in the author affiliations. The affiliations should appear as shown here:

Chuqian Chen^1,2^, Jiaxin Chen^1^, Guibing He^1^

**1** Department of Psychology and Behavioral Sciences, Zhejiang University, Hangzhou, P.R. China, **2** Department of Social Work and Social Administration, the University of Hong Kong, Pokfulam, Hong Kong, SAR, P.R. China.
