# Supplementary material for: Immorally obtained principal increases investors’ risk preference
Source: PLoS One. 2017 Apr 3;12(4):e0175181. doi: 10.1371/journal.pone.0175181 (PMC5378410; doi:10.1371/journal.pone.0175181)
Supplement: S1 File — (PDF) [file pone.0175181.s001.pdf]

S1

Reflective Moral Attentiveness Scale

Read the following statements and rate how much you agree with each of them with a number from 1 to 7. (1=strongly disagree, 7= strongly agree).

1. I regularly think about the ethical implications of my decisions.
2. I think about the morality of my actions almost every day.
3. I often find myself pondering about moral issues.
4. I often reflect on the moral aspects of my decisions.
5. I like to think about ethics.
