# Supplementary material for: Immorally obtained principal increases investors’ risk preference
Source: PLoS One. 2017 Apr 3;12(4):e0175181. doi: 10.1371/journal.pone.0175181 (PMC5378410; doi:10.1371/journal.pone.0175181)
Supplement: S2 File — (PDF) [file pone.0175181.s002.pdf]

S2

Bootstrap result in study 2

X = principal source morality

Y = investment preference

M = guilt

Total effect of X on Y					
Effect	SE	*t*	*p*	LLCI	ULCI
1.1382	.5313	2.1423	.0356	.0788	2.1976
Direct effect of X on Y					
Effect	SE	*t*	*p*	LLCI	ULCI
.6617	.5634	1.1744	.2442	-.4620	1.7853
Indirect effect of X on Y					
	Effect	Boot SE	BootLLCI	BootULCI	
Guilt	.4765	.2828	.0338	1.1126	

Bootstrap result in study 3a

X = principal source morality

Y = investment preference

M_1 = guilt, M_2= relative importance of "to reduce guilt"

Total effect of X on Y					
Effect	SE	*t*	*p*	LLCI	ULCI
1.3508	.5690	2.3738	.0176	.4148	2.2868
Direct effect of X on Y					
Effect	SE	*t*	*p*	LLCI	ULCI
1.2939	.6577	1.9675	.0491	.2122	2.3757
Indirect effect of X on Y					
	Effect	Boot SE	BootLLCI	BootULCI	
Total	.0712	.4184	-.6231	.7229	
Ind1	-.0102	.4225	-.6833	.6708	

Ind2	.0614	.1118	-.0439	.3194
Ind3	.0200	.1268	-.0906	.3369

Ind1 : principal source morality -> guilt -> investment choice

Ind2 : principal source morality -> guilt -> relative importance of "to reduce guilt" -> investment choice

Ind3 : principals source morality-> relative importance of "to reduce guilt"-> investment choice

X = principal source morality

Y = investment preference

M_1 = guilt, M_2= subjective value index

Total effect of X on Y					
Effect	SE	*t*	*p*	LLCI	ULCI
1.3508	.5690	2.3738	.0176	.2355	2.4661
Direct effect of X on Y					
Effect	SE	*t*	*p*	LLCI	ULCI
1.6355	.7228	2.2629	.0236	.2190	3.0521
Indirect effect of X on Y					
	Effect	Boot SE	BootLLCI	BootULCI	
Total	-.0818	.5085	-1.1828	.8038	
Ind1	-.2702	.4803	-1.3511	.5712	
Ind2	.3497	.2564	.0584	1.1900	
Ind3	-.1613	.2585	-.8522	.2365	

Ind1 :principal source morality ->guilt ->investment choice

Ind2 :principal source morality ->guilt ->subjective value index ->investment choice

Ind3 :principal source morality -> subjective value index -> investment choice

Bootstrap result in study 3b

X = principal source morality

Y = investment preference

M_1 = guilt, M_2= relative importance of "to reduce guilt"

Total effect of X on Y					
Effect	SE	*t*	*p*	LLCI	ULCI
1.1026	.4937	2.2335	.0255	.1350	2.0701
**Direct effect of X on Y**					
Effect	SE	*t*	*p*	LLCI	ULCI
1.0363	.6315	1.6409	.1008	-.2015	2.2740
**Indirect effect of X on Y**					
	Effect	Boot SE	BootLLCI	BootULCI	
Total	.0848	.4513	-.7496	1.0272	
Ind1	-.0413	.4695	-.9122	.9515	
Ind2	.1504	.1914	-.1309	.6335	
Ind3	-.0243	.1081	-.3949	.1069	

Ind1 : principal source morality -> guilt -> investment choice

Ind2 : principal source morality -> guilt -> relative importance of “to reduce guilt” -> investment choice

Ind3 : principals source morality-> relative importance of “to reduce guilt”-> investment choice

X = principal source morality

Y = investment preference

M_1 = guilt, M_2= subjective value index

Total effect of X on Y					
Effect	SE	*t*	*p*	LLCI	ULCI
1.1026	.4937	2.2335	.0255	.1350	2.0701
**Direct effect of X on Y**					
Effect	SE	*t*	*p*	LLCI	ULCI
1.2202	.6590	1.8365	.0663	-.0815	2.5017
**Indirect effect of X on Y**					
	Effect	Boot SE	BootLLCI	BootULCI	
Total	-.0316	.4623	-.9173	.9513	

Ind1	-.1741	.4467	-1.0321	.7805
Ind2	.2961	.2272	.0166	.9651
Ind3	-.1536	.2284	-.8566	.1213

Ind1 : principal source morality -> guilt -> investment choice

Ind2 : principal source morality -> guilt -> subjective value index -> investment choice

Ind3 : principal source morality -> subjective value index -> investment choice
