# Supplementary material for: Immorally obtained principal increases investors’ risk preference
Source: PLoS One. 2017 Apr 3;12(4):e0175181. doi: 10.1371/journal.pone.0175181 (PMC5378410; doi:10.1371/journal.pone.0175181)
Supplement: S3 File — (PDF) [file pone.0175181.s003.pdf]

S3

Minimum Data Set

Study 1

Participant	Gender	Age	Morality of description 1=immoral 2=moral	Principal's relevance to description 1=relevant 2=irrelevant	Interestingness of operation task	Investment choice 1=steady project 2=risky project
1	F	20	1	1	-1	2
2	F	23	1	1	-1	2
3	M	20	1	1	-1	2
4	F	21	1	1	-1	2
5	F	21	1	1	-1	1
6	M	19	1	1	-1	2
7	M	19	1	1	-1	2
8	F	20	1	1	-1	2
9	M	20	1	1	-2	1
10	F	23	1	1	-2	2
11	F	20	1	1	-2	2
12	M	20	1	1	-2	2
13	M	20	1	1	-2	2
14	F	20	1	1	-2	2
15	F	21	1	1	-2	2
16	F	23	1	1	-2	1
17	F	20	1	1	-2	1
18	M	19	1	1	-2	1
19	F	19	1	1	-3	2
20	M	20	1	1	-3	2
21	M	25	1	1	-3	2
22	M	20	1	1	-3	2
23	F	22	1	1	-4	1
24	M	22	2	1	-1	2
25	F	21	2	1	-1	1
26	F	19	2	1	-1	1
27	F	20	2	1	-1	1
28	F	21	2	1	-1	2
29	M	21	2	1	-1	2
30	M	24	2	1	-1	1
31	F	20	2	1	-2	1
32	M	27	2	1	-2	1
33	F	20	2	1	-2	1
34	F	18	2	1	-2	1
35	M	17	2	1	-2	1
36	F	20	2	1	-2	1
37	M	21	2	1	-3	2
38	F	22	2	1	-3	1
39	M	20	2	1	-3	2

Participant	Gender	Age	Morality of description<br>1=immoral<br>2=moral	Principal's relevance to description<br>1=relevant<br>2=irrelevant	Interestingness of operation task	Investment choice<br>1=steady project<br>2=risky project
40	F	20	2	1	-3	1
41	M	20	2	1	-3	1
42	F	20	2	1	-3	2
43	F	21	2	1	-3	2
44	F	21	2	1	-3	1
45	F	20	2	1	-3	1
46	M	21	2	1	-3	2
47	F	20	2	1	-3	1
48	F	18	2	1	-3	1
49	F	20	2	1	-4	1
50	M	20	2	1	-4	2
51	F	23	1	2	-2	2
52	F	23	1	2	-3	2
53	F	20	1	2	-1	2
54	F	20	1	2	-1	1
55	F	24	1	2	-1	1
56	M	23	1	2	-1	2
57	F	20	1	2	-2	1
58	F	22	1	2	-2	1
59	F	21	1	2	-2	2
60	M	20	1	2	-2	2
61	M	21	1	2	-2	2
62	F	20	1	2	-2	1
63	F	22	1	2	-2	2
64	F	22	1	2	-3	1
65	M	23	1	2	-3	2
66	F	20	1	2	-3	1
67	F	23	1	2	-3	1
68	M	24	1	2	-4	2
69	F	23	1	2	-2	2
70	F	23	1	2	-3	2
71	F	20	2	2	0	2
72	F	23	2	2	-2	2
73	F	20	2	2	-4	2
74	F	20	2	2	-2	2
75	F	23	2	2	-2	2
76	F	18	2	2	0	1
77	F	19	2	2	0	1
78	M	20	2	2	0	2
79	F	23	2	2	0	2
80	F	22	2	2	-1	1
81	M	23	2	2	-1	2
82	M	20	2	2	-1	1

Participant	Gender	Age	Morality of description<br>1=immoral<br>2=moral	Principal's relevance to description<br>1=relevant<br>2=irrelevant	Interestingness of operation task	Investment choice<br>1=steady project<br>2=risky project
83	F	20	2	2	-1	1
84	F	19	2	2	-1	1
85	M	23	2	2	-3	1
86	M	22	2	2	-3	2
87	F	20	2	2	-3	1
88	F	21	2	2	-3	2
89	M	21	2	2	-3	1
90	F	20	2	2	-3	1
91	F	20	2	2	-4	1
92	F	20	2	2	0	2
93	F	23	2	2	-2	2
94	F	20	2	2	-4	2
95	F	20	2	2	-2	2
96	F	23	2	2	-2	2
97	F	18	2	2	0	1

Study 2

Participant	Gender	Age	Principal source morality 1=immoral 2=moral	Interestingness of operation task	Preference for investment project (converted)	Angry	Guilty	Happy	Tired	Shy	Calm	Confident	Sad	Surprised	Anxious	Reflective moral attentiveness
1	F	20	1	-2	4	4	5	3	5	5	2	2	5	6	5	28
2	F	19	1	0	-2	1	1	1	2	1	7	2	1	3	1	25
3	M	17	1	0	-1	3	3	2	4	1	1	1	1	4	2	21
4	M	19	1	0	2	1	2	1	2	1	6	4	1	1	1	19
5	F	22	1	0	0	1	1	4	4	1	5	4	1	1	1	22
6	F	20	1	-2	-2	1	4	4	6	1	6	2	1	5	4	22
7	M	19	1	-1	2	1	4	4	4	4	3	3	3	3	3	26
8	F	24	1	-4	-2	2	6	1	5	6	1	1	4	5	4	12
9	F	20	1	-2	-2	1	2	2	2	1	4	1	1	2	2	24
10	F	25	1	-2	3	3	3	4	3	1	4	3	1	3	2	23
11	M	19	1	0	2	1	1	4	2	2	4	3	1	5	2	13
12	M	20	1	-1	-2	3	1	3	2	2	3	4	1	4	1	9
13	F	20	1	-3	0	3	7	1	6	6	1	1	2	7	7	17
14	M	21	1	-3	2	3	4	3	5	1	3	1	1	1	2	19
15	F	19	1	-2	1	4	6	3	5	5	2	1	3	4	5	17
16	M	17	1	-3	0	1	3	1	1	1	2	1	1	1	3	10
17	M	18	1	-1	1	3	3	5	5	4	4	3	3	4	5	25
18	F	24	1	0	1	1	5	4	4	2	5	2	2	4	4	22
19	M	24	1	-1	-4	1	1	2	1	1	4	4	1	1	1	18
20	M	27	1	-3	3	5	2	2	2	1	3	5	3	2	2	21
21	M	22	1	-3	-1	2	1	7	7	3	4	5	1	1	1	14
22	F	21	1	-2	-3	1	2	2	4	1	1	4	1	5	1	19
23	M	23	1	-3	-2	2	3	1	5	3	4	1	1	3	2	28
24	F	22	1	-3	2	4	1	3	5	2	4	4	5	5	5	12
25	M	22	1	-1	2	1	5	2	4	1	5	1	1	4	2	27
26	F	20	1	-4	3	1	7	1	4	1	4	4	4	4	3	19
27	F	18	1	-1	-3	1	2	2	2	4	5	3	1	3	3	25
28	F	20	1	-2	1	1	1	3	1	1	7	3	1	4	1	13
29	F	21	1	-2	-2	2	4	4	4	4	5	4	2	3	3	25

Participant	Gender	Age	Principal source morality 1=immoral 2=moral	Interestingness of operation task	Preference for investment project (converted)	Angry	Guilty	Happy	Tired	Shy	Calm	Confident	Sad	Surprised	Anxious	Reflective moral attentiveness
30	F	24	1	-3	-2	3	1	1	3	1	5	4	1	6	1	8
31	F	20	1	-2	-1	2	1	4	4	1	6	4	2	5	2	21
32	M	18	1	-1	2	1	5	3	1	1	4	3	4	3	2	19
33	F	21	1	-1	-3	3	2	4	5	3	4	2	4	5	5	25
34	M	19	1	-4	-3	2	6	1	4	2	1	1	2	1	6	24
35	F	20	1	-2	-3	1	1	2	2	1	4	4	1	3	1	22
36	F	20	1	-2	-1	4	6	1	6	2	3	2	3	4	6	19
37	M	18	1	-2	-2	1	2	3	4	1	5	3	1	1	2	22
38	F	18	1	-3	-2	3	5	1	6	5	2	2	1	3	4	24
39	F	26	1	-3	2	2	1	5	4	4	6	6	1	1	1	11
40	M	20	1	-1	-2	1	1	2	4	1	2	3	1	4	1	15
41	M	20	1	-1	-4	1	1	3	4	1	5	1	1	3	1	22
42	F	20	1	-3	1	4	5	3	6	4	5	2	4	4	4	21
43	F	20	2	-2	-4	1	1	3	4	1	5	4	1	1	1	23
44	F	22	2	-2	1	1	1	2	3	1	6	4	1	3	2	18
45	F	19	2	-3	-2	1	1	1	6	1	2	1	2	2	1	14
46	F	20	2	-2	-3	1	2	2	2	2	6	4	1	1	1	22
47	M	31	2	-1	-4	3	1	3	4	1	5	5	3	1	1	19
48	M	18	2	-1	-3	1	2	1	4	1	2	2	1	1	1	21
49	M	19	2	-1	1	1	1	1	4	1	5	4	1	1	1	19
50	F	21	2	-4	-4	1	1	2	7	1	4	1	1	5	1	22
51	F	20	2	-2	1	1	1	4	4	1	7	4	2	1	1	14
52	M	21	2	-2	-4	5	2	3	6	2	4	3	4	6	2	24
53	M	19	2	-3	-2	2	1	2	6	1	5	4	3	3	5	19
54	F	23	2	-2	-2	2	2	2	2	2	6	4	2	2	2	15
55	F	23	2	-2	1	1	1	4	4	1	7	4	1	2	1	31
56	M	22	2	-4	-3	1	1	1	4	1	5	2	1	1	1	25
57	M	23	2	-2	1	3	1	4	4	2	7	4	1	3	3	21
58	M	21	2	-2	-2	1	3	1	4	2	2	3	2	2	2	24
59	F	13	2	-4	-4	5	1	1	7	1	3	1	4	2	7	17

Participant	Gender	Age	Principal source morality 1=immoral 2=moral	Interestingness of operation task	Preference for investment project (converted)	Angry	Guilty	Happy	Tired	Shy	Calm	Confident	Sad	Surprised	Anxious	Reflective moral attentiveness
60	F	21	2	-2	2	1	1	1	4	1	4	1	1	1	2	11
61	F	20	2	-2	0	1	2	4	4	1	4	3	1	4	2	18
62	F	22	2	-2	-2	1	2	5	4	1	5	4	2	6	1	26
63	F	20	2	-1	-3	2	1	4	5	1	4	4	1	6	2	26
64	F	20	2	-1	-3	2	1	4	5	1	4	4	1	6	2	24
65	F	24	2	-3	-4	1	1	1	4	1	5	1	1	1	1	9
66	M	19	2	-4	-2	1	2	1	4	1	6	4	2	4	2	20
67	M	18	2	-2	1	2	1	4	3	3	5	4	1	2	2	23
68	F	21	2	-3	-3	1	1	2	6	1	5	1	2	1	1	19
69	F	24	2	-2	-2	4	1	4	4	1	5	4	1	1	1	21
70	F	21	2	-2	-2	1	1	1	6	1	5	1	1	3	1	23
71	M	22	2	-2	1	1	1	1	5	1	4	2	1	1	4	26
72	F	18	2	-2	1	1	1	2	5	1	5	1	1	1	1	15
73	F	22	2	-1	-3	1	1	4	2	1	3	4	1	1	1	9
74	M	20	2	-2	-2	1	2	3	4	3	4	4	1	5	4	22
75	F	20	2	-2	-2	1	1	3	4	1	4	3	1	1	1	18
76	F	19	2	-1	2	1	1	1	2	1	4	1	1	2	1	18
77	F	20	2	-2	-2	1	1	3	3	1	7	4	1	1	1	26
78	F	19	2	-1	-2	1	1	1	6	1	2	2	1	2	1	17
79	F	22	2	-3	2	4	1	3	6	1	5	1	1	4	4	17

Study 3a

Participant	Gender	Age	Principal source morality 1=immoral 2=moral	Guilty	Investment choice 0=steady project 1=risky project	N of pin	N of card case	N of paper cup	N of lollipop	Relative importance of "to ensure gains"	Relative importance of "to maximize gains"	Relative importance of "to reduce guilt"	Relative importance of "others"	Subjective value index
1	F	24	1	2	1	100	20	25	10	3	4	1	2	1.76
2	F	18	1	3	0	200	50	50	20	4	1	2	3	4.74
3	F	24	1	3	0	100	50	50	5	4	3	1	2	1.81
4	M	21	1	3	1	100	2	20	20	1	3	2	4	0.48
5	M	19	1	3	1	500	5	10	20	2	4	1	3	2.23
6	F	23	1	4	0	100	5	10	20	4	2	3	1	1
7	F	20	1	4	1	100	3	3	10	3	4	2	1	-1.04
8	M	20	1	5	1	100	5	10	5	3	4	1	2	-0.94
9	F	19	1	5	1	1000	2	200	10	2	3	1	4	3.08
10	M	26	1	5	1	100	5	5	10	3	4	1	2	-0.37
11	F	22	1	5	1	100	3	20	10	2	4	3	1	-0.03
12	F	22	1	6	0	20	10	10	20	4	2	3	1	0.31
13	F	21	1	6	0	100	10	100	1	3	1	2	4	0.27
14	M	20	1	6	1	40	6	1	20	3	4	2	1	-0.85
15	F	21	1	6	1	20	2	3	10	3	4	2	1	-2.8
16	F	27	1	6	1	3	2	1	3	2	4	3	1	-6.3
17	F	22	1	6	1	20	3	20	10	3	4	2	1	-1.3
18	M	28	1	6	1	10	2	20	5	4	3	2	1	-3.2
19	F	18	1	6	1	30	3	2	10	2	4	3	1	-2.22
20	F	20	1	7	0	100	10	10	10	4	3	2	1	0.57
21	F	23	1	7	0	200	10	2	4	4	1	3	2	-0.62
22	F	21	1	7	1	200	2	2	20	3	4	2	1	0.23
23	F	20	1	7	1	100	5	8	5	1	3	4	2	-1.17
24	F	23	1	7	1	50	10	30	5	3	4	2	1	-0.3
25	F	23	1	8	0	100	10	20	20	4	2	3	1	2.08
26	F	22	1	8	0	30	2	15	10	4	2	3	1	-1.67
27	F	18	1	8	0	10	2	50	10	4	3	2	1	-1.65
28	F	19	1	8	0	100	2	30	50	4	3	1	2	1.72
29	F	22	1	8	1	100	5	15	10	3	2	4	1	0.28

Participant	Gender	Age	Principal source morality 1=immoral 2=moral	Guilty	Investment choice 0=steady project 1=risky project	N of pin	N of card case	N of paper cup	N of lollipop	Relative importance of "to ensure gains"	Relative importance of "to maximize gains"	Relative importance of "to reduce guilt"	Relative importance of "others"	Subjective value index
30	M	19	1	8	1	100	10	2	10	1	2	4	3	-0.18
31	F	22	1	8	1	100	10	50	10	2	4	3	1	1.7
32	M	26	1	8	1	100	5	10	10	2	3	1	4	-0.02
33	M	20	1	9	0	40	4	2	10	4	2	3	1	-1.91
34	F	19	1	9	1	20	0	30	5	3	1	4	2	-3.44
35	F	22	2	1	0	1000	200	100	10	4	3	2	1	5.42
36	F	36	2	1	0	100	50	30	20	4	3	2	1	3.39
37	F	25	2	1	0	100	1000	100	10	4	3	2	1	4.31
38	F	23	2	1	0	100	20	1	20	4	3	2	1	0.76
39	F	21	2	1	0	100	10	1	20	4	3	1	2	0.24
40	F	20	2	1	0	1000	200	100	100	4	1	3	2	7.78
41	F	23	2	1	1	100	10	2	5	2	1	3	4	-1.1
42	F	22	2	2	0	100	5	1	4	4	3	1	2	-2.8
43	F	27	2	2	1	100	5	15	10	1	3	2	4	0.28
44	F	21	2	2	1	50	3	5	10	2	4	3	1	-1.6
45	F	21	2	3	0	100	5	50	20	4	3	1	2	2.13
46	F	25	2	3	0	100	20	10	10	4	1	3	2	1.09
47	M	25	2	3	0	100	0	50	10	3	2	2	1	-0.89
48	F	23	2	3	1	20	5	8	5	2	4	3	1	-2.44
49	F	24	2	4	0	100	5	5	20	4	2	1	3	0.65
50	F	24	2	4	0	100	10	15	10	4	3	2	1	0.86
51	F	28	2	4	0	200	20	80	20	4	3	2	1	4.64
52	F	22	2	4	0	20	15	3	5	4	3	1	2	-1.79
53	M	25	2	4	1	1000	2	1	3	1	3	2	4	-2.46
54	M	25	2	4	1	2	20	2	5	2	4	3	1	-3.2
55	F	39	2	5	0	20	25	20	6	4	2	3	1	-0.04
56	M	26	2	5	0	4	4	1	7	4	1	1	3	-4.3
57	F	22	2	5	0	100	10	1	20	4	3	1	2	0.24
58	M	25	2	5	0	100	2	12	1	3	2	1	4	-2.96
59	F	27	2	5	1	20	5	20	10	2	4	3	1	-0.78

Participant	Gender	Age	Principal source morality 1=immoral 2=moral	Guilty	Investment choice 0=steady project 1=risky project	N of pin	N of card case	N of paper cup	N of lollipop	Relative importance of "to ensure gains"	Relative importance of "to maximize gains"	Relative importance of "to reduce guilt"	Relative importance of "others"	Subjective value index
60	M	25	2	5	1	100	30	10	20	3	4	2	1	2.42
61	M	22	2	5	1	100	5	50	10	3	4	1	2	1.11
62	F	21	2	6	0	100	4	1	5	4	3	2	1	-2.63
63	F	27	2	6	0	100	10	50	20	4	2	3	1	2.71
64	F	30	2	6	0	10	2	10	10	4	3	2	1	-2.78
65	M	39	2	6	1	20	20	50	5	2	3	1	4	0.03
66	F	21	2	7	0	100	5	10	15	4	1	2	3	0.54
67	F	23	2	7	1	30	2	5	10	3	4	1	2	-2.31
68	M	24	2	7	1	30	10	50	10	2	1	3	4	0.77

Study 3b

Participant	Gender	Age	Principal source morality 1=immoral 2=moral	Guilty	Investment choice 0=steady project 1=risky project	N of pin	N of card case	N of paper cup	N of lollipop	Relative importance of "to ensure gains"	Relative importance of "to maximize gains"	Relative importance of "to reduce guilt"	Relative importance of "others"	Subjective value index
1	M	25	1	2	1	20	1	50	20	3	4	2	1	-1.47
2	F	23	1	3	0	100	50	30	10	4	3	2	1	2.27
3	F	21	1	4	1	100	10	50	10	2	3	1	4	1.54
4	F	23	1	4	1	100	5	2	6	1	4	3	2	-1.94
5	M	23	1	6	1	10	3	100	10	3	4	2	1	-1.05
6	F	23	1	6	0	100	4	30	20	4	3	2	1	1.07
7	F	19	1	7	1	100	5	50	3	2	3	1	4	-0.76
8	F	21	1	7	0	100	10	20	10	4	2	1	3	0.76
9	F	19	1	7	0	100	3	20	20	4	2	1	3	0.32
10	F	22	1	7	1	200	10	50	10	3	4	2	1	2.58
11	F	19	1	7	1	50	3	10	10	3	2	4	1	-1.89
12	M	22	1	7	0	50	10	20	10	4	2	3	1	-0.1
13	M	23	1	7	0	20	10	1	5	4	2	2	1	-3.71
14	M	22	1	8	1	100	4	20	50	3	4	2	1	1.66
15	M	22	1	8	1	100	20	25	20	3	4	1	2	2.59
16	F	21	1	8	1	10	2	2	10	3	4	2	1	-4.54
17	M	23	1	8	1	100	3	50	100	2	3	1	4	2.79
18	M	22	1	8	1	30	3	10	10	2	4	1	3	-2.16
19	M	26	1	8	1	50	10	100	20	2	4	2	3	2.17
20	M	22	1	8	1	20	5	25	10	1	3	4	2	-1.01
21	F	22	1	8	0	100	2	5	4	4	3	2	1	-3.28
22	M	18	1	8	0	100	5	20	20	4	2	3	1	0.93
23	M	23	1	9	1	100	10	20	20	2	3	4	1	1.59
24	M	23	1	9	1	100	1	3	5	2	3	1	1	-3.65
25	M	19	1	9	0	20	10	20	10	4	1	3	2	-0.69
26	F	18	1	9	0	100	20	50	20	4	3	2	1	3.04
27	F	23	1	9	1	100	10	10	5	1	2	4	3	-0.66
28	F	20	1	9	1	20	10	10	5	2	3	4	1	-2.11
29	F	24	1	9	1	100	5	2	5	3	4	4	1	-2.28

Participant	Gender	Age	Principal source morality 1=immoral 2=moral	Guilty	Investment choice 0=steady project 1=risky project	N of pin	N of card case	N of paper cup	N of lollipop	Relative importance of "to ensure gains"	Relative importance of "to maximize gains"	Relative importance of "to reduce guilt"	Relative importance of "others"	Subjective value index
30	F	18	1	9	0	100	10	50	50	4	3	1	2	3.39
31	F	22	1	9	0	10	2	5	3	4	1	3	2	-5.55
32	F	19	1	9	1	10	5	2	5	1	2	4	3	-4.27
33	F	21	1	9	1	200	3	1	5	2	4	3	1	-2.49
34	M	25	1	9	1	100	50	3	50	3	4	2	1	2.56
35	M	22	1	2	1	50	30	50	20	3	4	1	2	2.42
36	M	24	2	2	0	100	100	5	10	4	3	1	2	1.42
37	M	20	2	8	0	30	1	15	10	4	1	2	3	-3.07
38	M	21	2	6	1	100	3	1	10	3	4	2	1	-2.63
39	M	27	2	2	0	100	10	50	20	4	2	1	3	2.36
40	F	20	2	2	0	100	10	5	5	4	3	2	1	-0.99
41	F	22	2	2	0	50	20	20	30	4	3	1	2	2.04
42	F	26	2	2	0	100	10	100	10	4	3	1	2	2.21
43	F	21	2	2	1	200	10	15	20	2	4	1	3	2.31
44	F	23	2	1	0	100	10	100	10	4	3	1	2	2.21
45	F	22	2	7	0	500	10	20	50	4	3	1	2	4.03
46	F	17	2	7	0	100	10	2	20	4	1	2	3	0.1
47	F	21	2	3	0	25	3	50	20	4	3	2	1	-0.12
48	F	19	2	4	0	100	100	20	20	4	1	2	3	3.09
49	F	21	2	5	0	50	5	10	10	4	1	2	3	-1.27
50	F	19	2	6	0	100	3	20	7	4	3	2	1	-1
51	M	20	2	1	0	1000	10	200	20	4	2	1	3	5.7
52	F	20	2	6	1	20	10	20	10	2	3	1	4	-0.69
53	F	20	2	6	0	100	5	3	5	4	2	3	1	-1.92
54	F	20	2	5	1	50	10	5	3	2	3	1	4	-2.6
55	M	19	2	8	0	100	3	20	10	4	2	3	1	-0.51
56	M	22	2	3	1	50	10	30	6	3	4	2	1	-0.23
57	M	22	2	4	1	200	5	100	30	3	4	1	2	4.06
58	M	18	2	7	0	100	5	20	10	4	1	3	2	0.11
59	F	22	2	7	1	3	1	3	2	3	4	2	1	-7.64

Participant	Gender	Age	Principal source morality 1=immoral 2=moral	Guilty	Investment choice 0=steady project 1=risky project	N of pin	N of card case	N of paper cup	N of lollipop	Relative importance of "to ensure gains"	Relative importance of "to maximize gains"	Relative importance of "to reduce guilt"	Relative importance of "others"	Subjective value index
60	M	26	2	1	1	100	2	2	10	3	4	1	2	-2.54
61	M	26	2	2	0	500	100	50	30	4	3	1	2	5.93
62	M	18	2	5	1	50	3	5	20	2	4	3	1	-1.4
63	F	18	2	6	1	100	3	20	20	2	4	3	1	0.32
64	M	19	2	5	1	100	5	10	10	2	4	3	1	-0.41
65	M	19	2	5	1	100	5	5	10	1	4	3	2	-0.74
66	M	21	2	1	1	100	3	50	10	1	4	2	3	0.27
67	F	22	2	2	0	50	20	20	20	4	3	1	2	1.4
68	F	21	2	2	1	20	5	10	5	3	4	2	1	-2.77
69	F	22	2	1	0	100	2	10	10	4	1	3	2	-1.58
70	M	26	2	3	0	200	5	10	20	4	3	2	1	1.45
71	F	20	2	1	0	1000	20	100	5	4	3	2	1	3.85
